# Vine-Twining Inclusion Behavior of Amylose towards Hydrophobic Polyester, Poly(β-propiolactone), in Glucan Phosphorylase-Catalyzed Enzymatic Polymerization

**DOI:** 10.3390/life13020294

**Published:** 2023-01-20

**Authors:** Masa-aki Iwamoto, Jun-ichi Kadokawa

**Affiliations:** Graduate School of Science and Engineering, Kagoshima University, 1-21-40 Korimoto, Kagoshima 890-0065, Japan

**Keywords:** amylose, emulsion system, enzymatic polymerization, glucan phosphorylase, hydrophobic polyester, inclusion complex, poly(β-propiolactone), vine-twining process

## Abstract

This study investigates inclusion behavior of amylose towards, poly(β-propiolactone) (PPL), that is a hydrophobic polyester, via the vine-twining process in glucan phosphorylase (GP, isolated from thermophilic bacteria, *Aquifex aeolicus* VF5)-catalyzed enzymatic polymerization. As a result of poor dispersibility of PPL in sodium acetate buffer, the enzymatically produced amylose by GP catalysis incompletely included PPL in the buffer media under the general vine-twining polymerization conditions. Alternatively, we employed an ethyl acetate–sodium acetate buffer emulsion system with dispersing PPL as the media for vine-twining polymerization. Accordingly, the GP (from thermophilic bacteria)-catalyzed enzymatic polymerization of an α-d-glucose 1-phosphate monomer from a maltoheptaose primer was performed at 50 °C for 48 h in the prepared emulsion to efficiently form the inclusion complex. The powder X-ray diffraction profile of the precipitated product suggested that the amylose-PPL inclusion complex was mostly produced in the above system. The ^1^H NMR spectrum of the product also supported the inclusion complex structure, where a calculation based on an integrated ratio of signals indicated an almost perfect inclusion of PPL in the amylosic cavity. The prevention of crystallization of PPL in the product was suggested by IR analysis, because it was surrounded by the amylosic chains due to the inclusion complex structure.

## 1. Introduction

Amylose, which is a component of starch, is an energy storage polysaccharide in nature [1]. Because amylose comprises a left-handed helical conformation due to its chain structure composed of α(1→4)-linked repeating glucose (G) units [2,3], resulting in the double helix formation, it also acts as a host compound to construct supramolecular helical inclusion complexes with guest substrates with appropriate size and geometry, called V-amylose [4]. A hydrophobic cavity is created inside the helix, owing to the presence of hydroxy groups in the G units on the outer of the helix. Accordingly, hydrophobic monomeric and oligomeric molecules, such as fatty acids, have been included by amylose by direct hydrophobic interaction as driving force. We have considered polymeric guests as potential candidates for inclusion by amylose, compared to small guests, to show new functions and properties from supramolecular products [5]. However, only a few studies have been achieved on direct inclusion complexation of the long polymeric guests in the amylosic cavity by hydrophobic interaction [6,7,8,9,10]. The principal difficulty to directly interact long polymeric chains into the amylosic cavity is arising from such weak hydrophobic interaction as the driving force for the complexation.

Amylose with regularly controlled regio- and stereo-arrangements can be obtained by glucan phosphorylase (GP)-catalyzed enzymatic polymerization of α-d-glucose 1-phosphate (G-1-P) as a monomer [11,12,13,14], as enzymes have been identified as powerful biocatalysts for the highly controlled production of polysaccharides and the related materials [15,16,17,18]. To initiate the GP-catalyzed polymerization, a maltooligosaccharide primer is required in the reaction system and the initiation and propagation occur at the nonreducing end of the α(1→4)-glucan chain, according to the following primary reaction; [α(1→4)-G]*_n_* + G-1-P → [α(1→4)-G]*_n+1_* + inorganic phosphate. We have found that amylose, thus, enzymatically produced in the GP-catalyzed polymerization, includes polymeric guests with moderate hydrophobicity in its cavity, which are dispersible in the polymerization media, via the vine-twining process [19,20,21,22]. In the system, the enzymatically elongating amylosic chain gradually twines around the polymeric chains to directly construct amylosic supramolecular helical inclusion complexes with the polymers. The enzymatically elongation of the shorter α(1→4)-glucan, i.e., maltooligosaccharide, into the longer *α*(1→4)-glucan, i.e., amylose, is conceived to provide appropriate dynamic field for easier inclusion complexation towards the polymeric guests, compared to the direct inclusion into the amylosic cavity. Because of schematic similarity of the present system to the growing plant vines that gradually twine around a support rod, it has been named ‘vine-twining polymerization’ [19,20,21,22].

For example, slender polyesters with moderate hydrophobicity, e.g., poly(ε-caprolactone), poly(δ-valerolactone), and poly(γ-butyrolactone) (PCL, PVL, and PBL), that do not carry side substituents, can be employed as the polymeric guests to construct the corresponding amylosic helical inclusion complexes through vine-twining polymerization [23,24,25]. When vine-twining polymerization was attempted in the presence of the structural isomer of PBL with methyl substituents, i.e., poly[(*R*)-3-hydroxybutyrate] (PRHB), as the guest polyester, only its low molecular weight (approximately 500) oligomer showed ability for the formation of an inclusion complex with amylose under the following specific conditions in the GP-catalyzed enzymatic polymerization system [26]. The enzymatic polymerization using oligo[(*R*)-3-hydroxybutyrate] (ORHB) was first caried out by the thermostable GP (from *Aquifex aeolicus* VF5) catalysis at elevated temperature (at 70 °C), higher than that for the general vine-twining polymerization (at 45–50 °C), to obtain water-soluble short α(1→4)-glucan (amylosic oligomer, named single amylose without double helical assembly) [27], that interacts weakly with ORHB. The reaction mixture was then cooled to 45 °C over 7 h to incur the further chain-elongation from the single amylose by the GP catalysis, which twiningly complexed around ORHB to yield the amylose-ORHB inclusion complex. This system was achieved by using thermostable GP because its activity was retained at elevated temperature like 70 °C. Meanwhile, the other GP isolated from potato, the most widely used GP, which has been used in the previously reported vine-twining polymerization system using PCL and PVL [23,24], cannot be employed under such temperature conditions due to its instability at that temperature. In the other hydrophobic polyesters with methyl substituents, but comprising a lower number of carbons than that in PRHB leading to less hydrophobic, that is, poly(lactic acid) (PLA) stereoisomers depending on stereo-directions of methyl substituents (poly(l-lactic acid), poly(d-lactic acid), and poly(dl-lactic acid) (PLLA, PDLA, and PDLLA), an amylosic inclusion complex is obtained only from PLLA under the general vine-twining polymerization conditions [28]. The helical direction is critical whether amylose incudes PLAs or not by the vine-twining process, in which PLLA constructs a left handed helix, similar to that of amylose, while opposite and irregular helical structures are formed from PDLA and PDLLA, respectively [29]. PRHB also constructs a left handed helical conformation, leading to the inclusion ability of its oligomer by amylose. Poly(glycolic acid) (PGA), which comprises the same main-chain structure to that in PLA, but without methyl substituents, constructs highly crystalline structure and shows poor dispersibility in aqueous media, leading to no formation of inclusion complexes with amylose [30]. The results above strongly suggest that hydrophobicity, bulkiness of polyesters, and dispersibility in aqueous media are representative factors that must be included in the amylosic cavity in vine-twining polymerization. Polyethers, that can be dispersible in aqueous media, attributable to their moderate hydrophobicity, such as poly(tetramethylene oxide) and poly(trimethylene oxide), have also been employed as the polymeric guests in vine-twining polymerization to construct the corresponding amylosic helical inclusion complexes [31,32,33]. Both strongly hydrophobic and hydrophilic polyethers, such as poly(hexamethylene oxide) and poly(ethylene oxide) have undergone strongly aggregate in aqueous media, and weak hydrophobic interactions, respectively, to not form amylosic inclusion complexes in vine-twining polymerization [32]. Furthermore, the vine-twining polymerization system has been extended to fabricating macroscopic supramolecular materials with a larger scale, such as supramolecular hydrogels, using polymeric primers, where the G_7_ primers are covalently immobilized on appropriate polymeric substrates [5,22].

**Figure 1:**
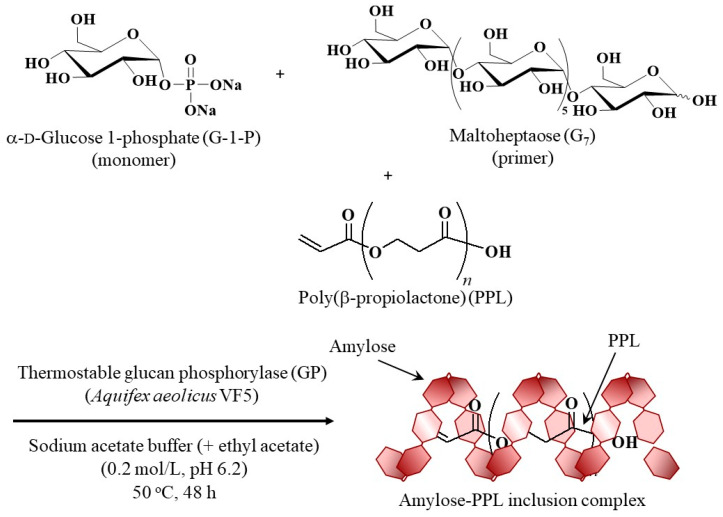
Vine-twining polymerization using poly(β-propiolactone) (PPL) as guest polyester.

In this study, we investigate the inclusion behavior of amylose towards poly(β-propiolactone) (PPL), which is the structural isomer of PLA in the absence of methyl substituents, as a new guest polyester in vine-twining polymerization (Figure 1). PPL was prepared by lipase-catalyzed enzymatic ring-opening polymerization of β-propiolactone (PL) as a monomer according to the literature procedure [34]. Owing to its highly crystalline nature and low dispersibility in aqueous media, the GP-catalyzed enzymatic polymerization system using PPL in sodium acetate buffer did not sufficiently result in its inclusion in the amylosic cavity by the vine-twining manner. On the other hand, when vine-twining polymerization was attempted by the GP-catalyzed enzymatic polymerization under emulsion conditions, constructed from ethyl acetate/sodium acetate buffer/PPL, an amylose-PPL inclusion complex was obtained. The product was characterized by X-ray diffraction (XRD), ^1^H NMR, and IR analysis. The present study develops the new emulsion system with dispersing the guest polymer for vine-twining polymerization, which accelerate inclusion ability of the enzymatically produced amylose to obtain amylosic supramolecular inclusion complexes.

## 2. Materials and Methods

### 2.1. Materials

GP, isolated from thermophilic bacteria (from *Aquifex aeolicus* VF5), was kindly supplied from Dr. Takeshi Takaha (Sanwa Starch Co., LTD., Nara, Japan) [35]. Lipase from *Pseudomonas fluorescens* was purchased from Sigma-Aldrich, Darmstadt, Germany). G_7_ was synthesized by selective hydrolysis of a glycosidic bond in β-cyclodextrin under acidic conditions [36]. The unreacted β-cyclodextrin was removed from the reaction mixture after inclusion complexation with *p*-xylene. All other reagents and solvents were available commercially and used as received.

### 2.2. Preparation of PPL

The guest polyester, PPL, was synthesized by enzymatic ring-opening polymerization of PL catalyzed by lipase according to the literature procedure [34]. Mixtures of PL (0.500–0.526 g, 6.93–7.30 mmol) with lipase (200–212U) was heated at 80 °C for 6–24 h with stirring under argon. After chloroform (20 mL) was added to the reaction mixtures, the precipitated lipase was removed by filtration and the filtrate was concentrated. Water was added to the concentrated solution to precipitate the products, which were isolated by centrifugation and lyophilized to give PPL (0.0915–0.120 g); ^1^H NMR (CDCl_3_, Figure S1) δ 2.63–2.71 (br s, -CH_2_C=O), 4.30–4.46 (br s, -CH_2_OC=O), 5.83–5.85, 6.07–6.14, 6.38–6.43 (m, CH_2_=CHC=O (terminal)). The *M*_n_ values were calculated from integrated ratios of the main-chain signals to the terminal signals to be 1360–2230 (depending on reaction times).

### 2.3. Vine-Twining Polymerization

To a suspension of PBL (*M*_n_ = 2230, 0.0646 g) with ethyl acetate (2.0 mL), which was prepared by ultrasonication of the mixture, was added sodium acetate buffer (0.2 mol/L, pH 6.2, 10 mL) and the mixture was ultrasonicated to form an emulsion. After G-1-P (0.218 g, 0.84 mmol), G_7_ (0.0037 g, 0.003 mmol), and thermostable GP (15 U) were added to the emulsion, the obtained mixture was maintained at 50 °C for 48 h with vigorously stirring. The product, precipitated, was removed by filtration, washed successively with water, acetone, and chloroform, and dried in vacuo to obtain the inclusion complex (0.0565 g). ^1^H NMR (DMSO-*d*_6_) δ 2.61–2.64 (br s, -CH_2_C=O), 3.33–3.96 (br m, H2-H6, overlapping with HOD), 4.12–4.26 (br s, -CH_2_OC=O), 4.58, 5.41, 5.55 (br s, -OH), 5.09 (br s, H1).

### 2.4. Measurements

Powder XRD measurements were conducted using a Rigaku Geigerflex RADIIB diffractometer (PANalytical B.V., EA Almelo, the Netherlands) with Ni-filtered CuKα radiation (*λ* = 0.15418 nm). Laser microscopic images were obtained by a Keyence VK-8500 laser microscope (Keyence, Osaka, Japan). ^1^H NMR spectra were recorded on a JEOL ECX 400 spectrometer (JEOL, Akishima, Tokyo, Japan). IR spectra were recorded on a PerkinElmer Spectrum Two spectrometer (PerkinElmer Japan Co., Ltd., Yokohama, Japan).

## 3. Results and Discussion

Prior to performing vine-twining polymerization, we prepared PPL by lipase (from *Pseudomonas fluorescens*)-catalyzed enzymatic ring-opening polymerization of PL at 80 °C for 6–24 h according to the literature procedure [34]. The structure of the product was supported by the ^1^H NMR spectrum to be PPL (Figure S1 and the data in Section 2.2). The signals assigned to a terminal acrylate group were detected at around δ 5.8–6.5, which was derived by dehydration of the terminal β-hydroxypropionate group, probably occurring during isolation procedures. The *M*_n_ values of the products were estimated by ^1^H NMR analysis to be 1360–2230, depending on reaction times.

**Figure 2:**
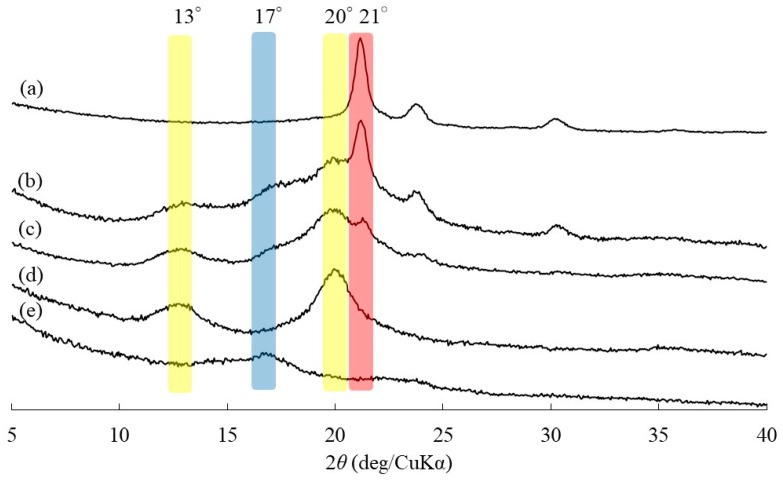
Powder XRD profiles of (a) PPL, (b) product obtained by vine-twining polymerization in sodium acetate buffer, (c) product obtained by vine-twining polymerization in ethyl acetate–sodium acetate buffer emulsion system, (d) amylose-poly(γ-butyrolactone) (PBL) inclusion complex, (e) double helical amylose.

**Figure 3:**
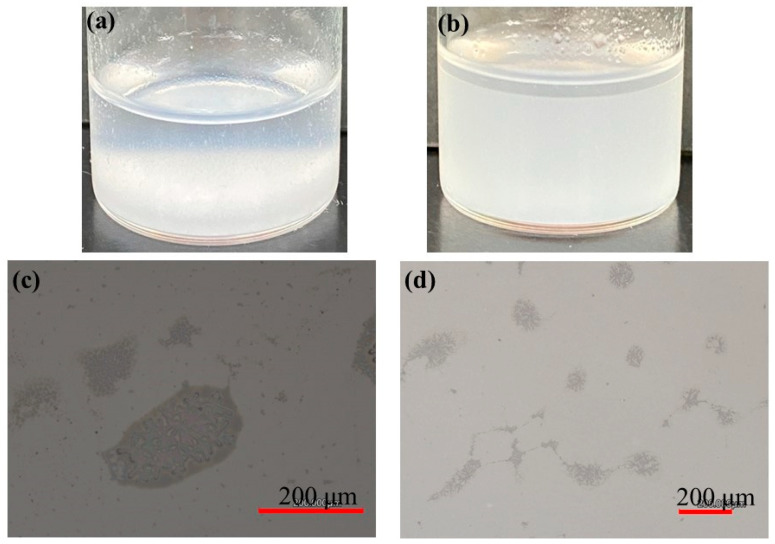
Photographs and laser microscopic images of (**a**,**c**) mixture of PPL with sodium acetate buffer after ultrasonication and (**b**,**d**) mixture of PPL with ethyl acetate/sodium acetate buffer after ultrasonication.

Vine-twining polymerization using PPL (*M*_n_ = 1360) as the guest polyester was then attempted by the procedure, the same as that, which previously used PPL, PVL, and PCL, as follows (Figure 1) [23,24,25]. After PPL was dispersed in sodium acetate buffer (0.2 mol/L, pH 6.2) by ultrasonication, the thermostable GP (from *Aquifex aeolicus* VF5)-catalyzed enzymatic polymerization of G-1-P from a maltoheptaose (G_7_) primer (molar ratio = 280:1) was conducted at 50 °C for 48 h in the resulting aqueous mixture. The powder XRD profile of the precipitated product (Figure 2b) slightly showed the peaks at 13 and 20° derived from the amylosic inclusion complex (with yellow shadows, corresponding to V-amylose crystalline structure), as observed in that of the previously reported amylose-PBL inclusion complex (Figure 2d) [25], but also exhibited the peaks at 17 and 21° ascribed to the crystalline structures of a double helical amylose and a pure PPL, respectively (with blue and red shadows), as shown in Figure 2a,e. The XRD result strongly indicated the occurrence of an incomplete inclusion of PPL in the amylosic cavity by the above experimental system. This incomplete inclusion was owing to the poor dispersibility of PPL in aqueous media, as agglomerates were observed in a mixture of PPL with sodium acetate buffer after ultrasonication (Figure 3a). The poor dispersibility of PPL is probably due to its highly crystalline nature, as quite sharp peaks are observed in the XRD profile of PPL (Figure 2a), which is similar to that of PGA. Indeed, the solubilities of PPL and PBL, with the structural difference of only a methylene group, in common organic solvents, are much different, where the former polyester is insoluble in acetone and ethyl acetate, while the latter polyester is soluble in such solvents.

To provide a well-dispersed PPL in buffer media, we then employed the following mixed solvent system. After PPL was suspended in ethyl acetate by ultrasonication, sodium acetate buffer was added. Subsequently, ultrasonication of the resulting mixture gave an emulsion-like system, as shown in Figure 3b. Indeed, the laser micrographic image of the produced system (Figure 3d) indicated the presence of emulsified droplets. The laser micrographic image of the mixture of PPL with sodium acetate buffer after ultrasonication in Figure 3c showed microparticle morphology, as well as large agglomerates. Accordingly, we speculated that such microparticles could act as stabilizer for the formation of the emulsion system.

Therefore, the thermostable GP-catalyzed enzymatic polymerization was carried out in the presence of G-1-P and G_7_ (molar ratio = 280:1) at 50 °C for 48 h in the emulsion system containing the dispersed PPL (*M*_n_ = 2230) for the progress of the vine-twining inclusion of amylose towards PPL (Figure 1). The product, precipitated, was separated by filtration, washed successively with water, acetone, and chloroform, and dried. The XRD profile of the product (Figure 2c) mostly detected peaks at 13 and 20° derived from the amylosic inclusion complex (with yellow shadows) and did not, largely, observe peaks at 17 and 21° assignable to the crystalline structures of a double helical amylose and a pure PPL, respectively (with blue and red shadows, Figure 2a,e). The XRD result suggested that vine-twining polymerization progressed well in the above emulsion system to obtain the amylose-PPL inclusion complex. The XRD pattern also suggested the formation of the 6_1_ amylosic helix in the product [37], which were the same conformation in the previously reported amylose-polyester inclusion complexes (e.g., Figure 2d for amylose-PBL inclusion complex) [25].

**Figure 4:**
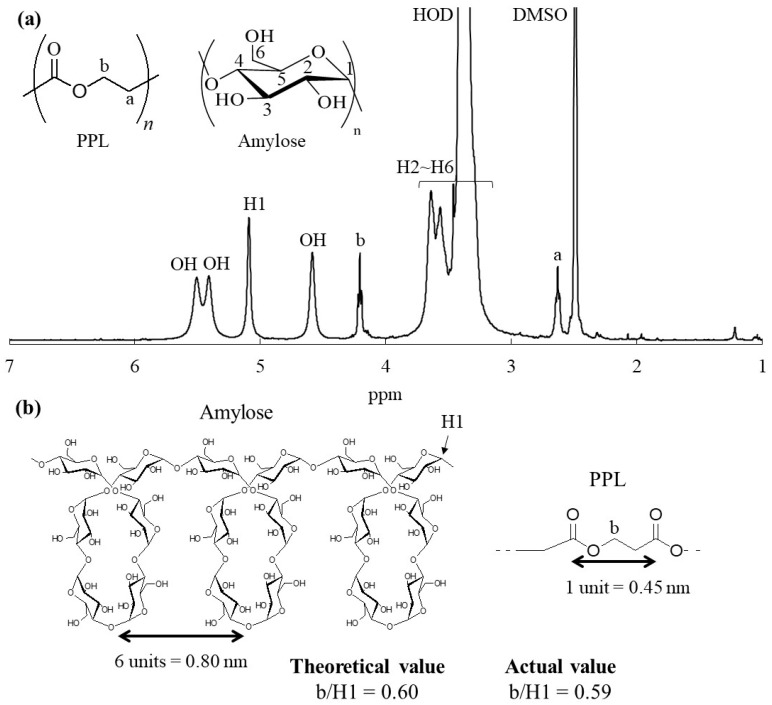
(**a**) ^1^H NMR spectrum of product in DMSO-*d*_6_ obtained by vine-twining polymerization in ethyl-acetate-sodium acetate buffer emulsion system and (**b**) illustration of repeat distance of 6_1_ amylosic helix and unit length of PPL.

**Figure 5:**
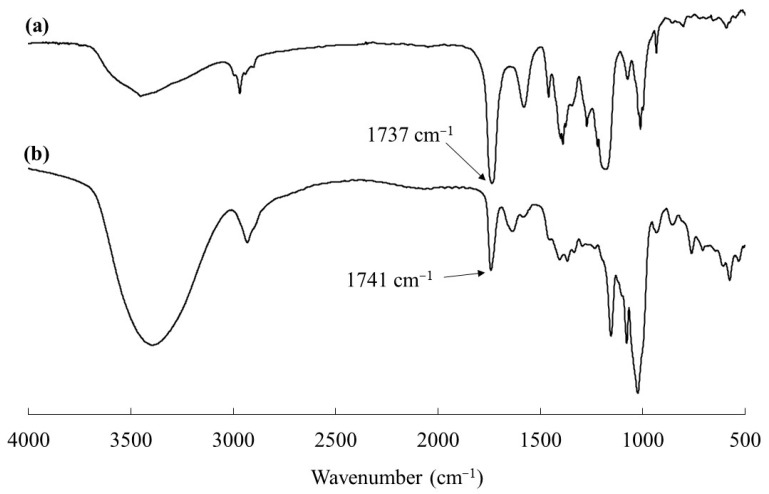
IR spectra of (**a**) PPL and (**b**) product obtained by vine-twining polymerization in ethyl-acetate-sodium acetate buffer emulsion system.

The ^1^H NMR spectrum of the product in DMSO-*d*_6_ also indicated its inclusion complex structure, because signals assigned not only to amylose, but also to PPL were detected as depicted in Figure 4a (the NMR data are described in Section 2.3). The repeat distance of the 6_1_ amylosic helix had been calculated to be 0.80 nm [38,39], whereas a PPL unit length was calculated for the present study to be 0.45 nm as shown in Figure 4b. Therefore, the length of one PPL unit corresponds to 3.38 repeating G units in the 6_1_ amylosic helix. On the basis of the above estimations, the theoretical integrated ratio of the amylosic H1 (anomeric) signal to the methylene signal (-CH_2_OC=O, b) of PPL (b/H1) in the ^1^H NMR profile of the ideal inclusion complex is calculated to be 0.60. The actual b/H1 value in the ^1^H NMR spectrum of the product (Figure 4a) was evaluated to be 0.59, suggesting a 99% inclusion ratio of PPL in the amylosic cavity. The inclusion complex structure of the product was also suggested by the IR analysis. The C=O absorption peak ascribable to ester linkage in the IR spectrum of a pure PPL with crystalline structure was observed at 1737 cm^−1^ (Figure 5a), that shifted to higher wavenumber at 1741 cm^−1^ in that of the vine-twining polymerization product (Figure 5b). The shift is owing to uncrystallization of PPL in the product because amylose chains have surrounded PPL by inclusion [24]. The uncrystallized structure of PPL in the vine-twining polymerization product was also supported by the XRD result as shown in Figure 2c, which did not, in the main, observe the diffraction peaks derived from the crystalline PPL. All of the results presented above revealed that vine-twining polymerization successfully progressed in the emulsion system, which was formed from PPL, ethyl acetate, and sodium acetate buffer, to produce the amylose–PPL inclusion complex.

## 4. Conclusions

In this study, we investigated the vine-twining inclusion behavior of amylose towards the hydrophobic polyester, PPL, in the thermostable GP-catalyzed enzymatic polymerization. As PPL was not sufficiently dispersed in sodium acetate buffer owing to its highly crystallinity, an ethyl acetate–sodium acetate buffer emulsion system with dispersing PPL was employed as the media for the acceleration of inclusion by the amylosic cavity in the GP-catalyzed enzymatic polymerization. The XRD, ^1^H NMR, and IR results of the precipitated product supported the structure of the amylose–PPL inclusion complex. From the integrated ratio of the signals in the ^1^H NMR spectrum, almost perfect inclusion of PPL by amylose was suggested. As this study has indicated that the present emulsion system strongly assists inclusion of PPL by amylose in the GP-catalyzed enzymatic polymerization, a similar system using other poorly dispersed polymers in aqueous media will be performed to obtain new amylosic supramolecular inclusion complexes in the future.

## Data Availability

Not applicable.

## Supplementary Materials

The following supporting information can be downloaded at: https://www.mdpi.com/article/10.3390/life13020294/s1, Figure S1: ^1^H NMR spectrum of poly(β-propiolactone) (PPL) in CDCl_3_.

## References

[1] Song E.H., Shang J., Ratner D.M., Matyjaszewski K., Möller M. (2012). Polysaccharides. Polymer Science: A Comprehensive Reference.

[2] Imberty A., Chanzy H., Perez S., Buleon A., Tran V. (1988). The double-helical nature of the crystalline part of A-starch. J. Mol. Biol..

[3] Imberty A., Perez S. (1988). A revisit to the three-dimensional structure of B-type starch. Biopolymers.

[4] Putseys J.A., Lamberts L., Delcour J.A. (2010). Amylose-inclusion complexes: Formation, identity and physico-chemical properties. J. Cereal. Sci..

[5] Kadokawa J., Thakur K.V., Thakur K.M. (2015). Hierarchically fabrication of amylosic supramolecular nanocomposites by means of inclusion complexation in phosphorylase-catalyzed enzymatic polymerization field. Eco-Friendly Polymer Nanocomposites: Processing and Properties.

[6] Shogren R.L., Greene R.V., Wu Y.V. (1991). Complexes of starch polysaccharides and poly(ethylene coacrylic acid)—Structure and stability in solution. J. Appl. Polym. Sci..

[7] Shogren R.L. (1993). Complexes of starch with telechelic poly(e-caprolactone) phosphate. Carbohydr. Polym..

[8] Rachmawati R., Woortman A.J.J., Loos K. (2013). Facile preparation method for inclusion complexes between amylose and polytetrahydrofurans. Biomacromolecules.

[9] Rachmawati R., Woortman A.J.J., Loos K. (2013). Tunable properties of inclusion complexes between amylose and polytetrahydrofuran. Macromol. Biosci..

[10] Rachmawati R., Woortman A.J.J., Loos K. (2014). Solvent-responsive behavior of inclusion complexes between amylose and polytetrahydrofuran. Macromol. Biosci..

[11] Ziegast G., Pfannemuller B. (1987). Linear and star-shaped hybrid polymers.4. Phosphorolytic syntheses with di-functional, oligo-functional and multifunctional primers. Carbohydr. Res..

[12] Fujii K., Takata H., Yanase M., Terada Y., Ohdan K., Takaha T., Okada S., Kuriki T. (2003). Bioengineering and application of novel glucose polymers. Biocatal. Biotransform..

[13] Yanase M., Takaha T., Kuriki T. (2006). a-Glucan phosphorylase and its use in carbohydrate engineering. J. Sci. Food Agric..

[14] Kadokawa J. (2017). a-Glucan phosphorylase: A useful catalyst for precision enzymatic synthesis of oligo- and polysaccharides. Curr. Org. Chem..

[15] Kadokawa J. (2011). Precision polysaccharide synthesis catalyzed by enzymes. Chem. Rev..

[16] Shoda S., Uyama H., Kadokawa J., Kimura S., Kobayashi S. (2016). Enzymes as green catalysts for precision macromolecular synthesis. Chem. Rev..

[17] Kadokawa J. (2020). α-glucan phosphorylase-catalyzed enzymatic reactions to precisely synthesize non-natural polysaccharides. ACS Symposium Series.

[18] Kadokawa J. (2022). Glucan phosphorylase-catalyzed enzymatic synthesis of unnatural oligosaccharides and polysaccharides using nonnative substrates. Polym. J..

[19] Kadokawa J. (2012). Preparation and applications of amylose supramolecules by means of phosphorylase-catalyzed enzymatic polymerization. Polymers.

[20] Kadokawa J. (2013). Architecture of amylose supramolecules in form of inclusion complexes by phosphorylase-catalyzed enzymatic polymerization. Biomolecules.

[21] Orio S., Shoji T., Yamamoto K., Kadokawa J. (2018). Difference in macroscopic morphologies of amylosic supramolecular networks depending on guest polymers in vine-twining polymerization. Polymers.

[22] Kadokawa J. (2020). Synthesis of amylosic supramolecular materials by glucan phosphorylase-catalyzed enzymatic polymerization according to the vine-twining approach. Synlett.

[23] Kadokawa J., Kaneko Y., Nakaya A., Tagaya H. (2001). Formation of an amylose-polyester inclusion complex by means of phosphorylase-catalyzed enzymatic polymerization of a-D-glucose 1-phosphate monomer in the presence of poly(e-caprolactone). Macromolecules.

[24] Kadokawa J., Nakaya A., Kaneko Y., Tagaya H. (2003). Preparation of inclusion complexes between amylose and ester-containing polymers by means of vine-twining polymerization. Macromol. Chem. Phys..

[25] Iwamoto M., Watanabe R., Yamamoto K., Kadokawa J. (2022). Inclusion behavior of amylose toward hydrophobic polyester, poly(γ-butyrolactone), in vine-twining polymerization. Colloid Polym. Sci..

[26] Kadokawa J., Wada Y., Yamamoto K. (2021). Preparation of amylose-oligo[(R)-3-hydroxybutyrate] inclusion complex by vine-twining polymerization. Molecules.

[27] Kadokawa J., Orio S., Yamamoto K. (2019). Formation of microparticles from amylose-grafted poly(γ-glutamic acid) networks obtained by thermostable phosphorylase-catalyzed enzymatic polymerization. RSC Adv..

[28] Kaneko Y., Ueno K., Yui T., Nakahara K., Kadokawa J. (2011). Amylose's recognition of chirality in polylactides on formation of inclusion complexes in vine-twining polymerization. Macromol. Biosci..

[29] Tanaka T., Sasayama S., Yamamoto K., Kimura Y., Kadokawa J. (2015). Evaluating relative chain orientation of amylose and poly(L-lactide) in inclusion complexes formed by vine-twining polymerization using primer-guest conjugates. Macromol. Chem. Phys..

[30] Nomura S., Kyutoku T., Shimomura N., Kaneko Y., Kadokawa J. (2011). Preparation of inclusion complexes composed of amylose and biodegradable poly(glycolic acid-co-ɛ-caprolactone) by vine-twining polymerization and their lipase-catalyzed hydrolysis behavior. Polym. J..

[31] Kadokawa J., Kaneko Y., Tagaya H., Chiba K. (2001). Synthesis of an amylose-polymer inclusion complex by enzymatic polymerization of glucose 1-phosphate catalyzed by phosphorylase enzyme in the presence of polyTHF: A new method for synthesis of polymer-polymer inclusion complexes. Chem. Commun..

[32] Kadokawa J., Kaneko Y., Nagase S., Takahashi T., Tagaya H. (2002). Vine-twining polymerization: Amylose twines around polyethers to form amylose—Polyether inclusion complexes. Chem. Eur. J..

[33] Kaneko Y., Beppu K., Kadokawa J. (2009). Amylose Selectively Includes a Specific Range of Molecular Weights in Poly(tetrahydrofuran)s in Vine-Twining Polymerization. Polym. J..

[34] Matsumura S., Beppu H., Tsukada K., Toshima K. (1996). Enzyme-catalyzed ring-opening polymerization of β-propiolactone. Biotechnol. Lett..

[35] Bhuiyan S.H., Rus’d A.A., Kitaoka M., Hayashi K. (2003). Characterization of a hyperthermostable glycogen phosphorylase from *Aquifex aeolicus* expressed in *Escherichia coli*. J. Mol. Catal. B Enzym..

[36] von Braunmühl V., Jonas G., Stadler R. (1995). Enzymatic grafting of amylose from poly(dimethylsiloxanes). Macromolecules.

[37] Yamashita Y. (1965). Single crystals of amylose V complexes. J. Polym. Sci. Part A.

[38] Zobel H.F., French A.D., Hinkle M.E. (1967). X-Ray diffraction of oriented amylose fibers. II. Structure of V amyloses. Biopolymers.

[39] Zobel H.F. (1988). Starch crystal transformations and their industrial importance. Starch—Stärke.

