# Supplementary material for: Vine-Twining Inclusion Behavior of Amylose towards Hydrophobic Polyester, Poly(β-propiolactone), in Glucan Phosphorylase-Catalyzed Enzymatic Polymerization
Source: Life (Basel). 2023 Jan 20;13(2):294. doi: 10.3390/life13020294 (PMC9958898; doi:10.3390/life13020294)
Supplement: Supplementary file 1 [file life-13-00294-s001.zip › life-2154411-supplementary.pdf]

Supporting Information

Vine-twining Inclusion Behavior of Amylose Towards Hydrophobic Polyester, Poly(β-propiolactone), in Glucan Phosphorylase-catalyzed Enzymatic Polymerization

Masa-aki Iwamoto[a] and Jun-ichi Kadokawa[a,*]

[a] Graduate School of Science and Engineering, Kagoshima University, 1-21-40 Korimoto, Kagoshima 890-0065, Japan

*E-mail : kadokawa@eng.kagoshima-u.ac.jp ; Tel.: +81-99-285-7743

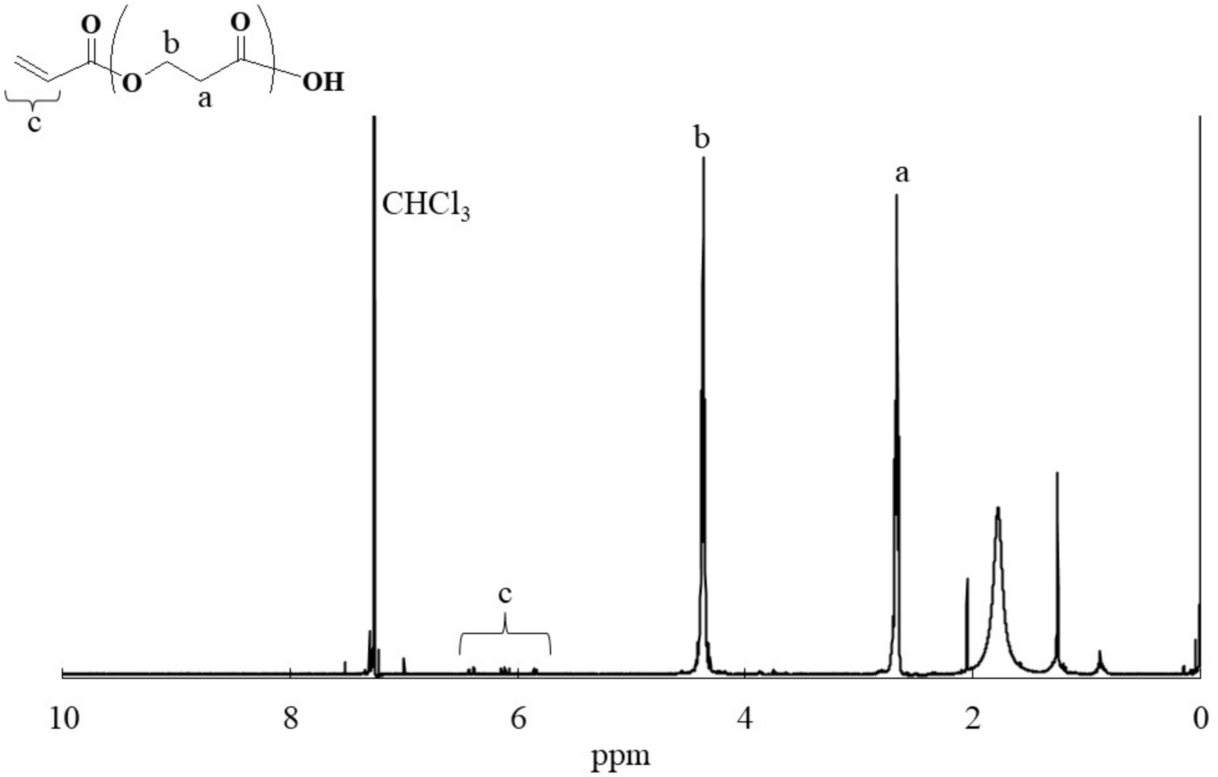

Figure S1. ^{1}H NMR spectrum of poly(β-propiolactone) (PPL) in $CDCl_3$.
